# Logomaker: beautiful sequence logos in Python

**DOI:** 10.1093/bioinformatics/btz921

**Published:** 2019-12-10

**Authors:** Ammar Tareen, Justin B Kinney

**Affiliations:** Simons Center for Quantitative Biology, Cold Spring Harbor Laboratory, Cold Spring Harbor, NY 11724, USA

## Abstract

**Summary:**

Sequence logos are visually compelling ways of illustrating the biological properties of DNA, RNA and protein sequences, yet it is currently difficult to generate and customize such logos within the Python programming environment. Here we introduce Logomaker, a Python API for creating publication-quality sequence logos. Logomaker can produce both standard and highly customized logos from either a matrix-like array of numbers or a multiple-sequence alignment. Logos are rendered as native matplotlib objects that are easy to stylize and incorporate into multi-panel figures.

**Availability and implementation:**

Logomaker can be installed using the pip package manager and is compatible with both Python 2.7 and Python 3.6. Documentation is provided at http://logomaker.readthedocs.io; source code is available at http://github.com/jbkinney/logomaker.

## 1 Introduction

Sequence logos provide evocative graphical representations of the functional properties of DNA, RNA and protein sequences. Logos consist of characters stacked upon one another at a series of integer-valued positions, with the height of each character conveying some type of information about its biological importance. This graphical representation was introduced by Schneider and Stephens (1990) for illustrating statistical properties of multiple-sequence alignments. Although the specific representation they advocated is still widely used, sequence logos have since evolved into a general data visualization strategy that can be used to illustrate many different kinds of biological information (Kinney and McCandlish, 2019). For example, logos can be used to illustrate base-pair-specific contributions to protein–DNA binding energy (Foat *et al.*, 2006), the effects of mutations in massively parallel selection experiments, [Bibr btz921-B14] and attribution method visualizations of deep neural networks (Jaganathan *et al.*, 2019; Shrikumar *et al.*, 2017).

**Fig. 1.**
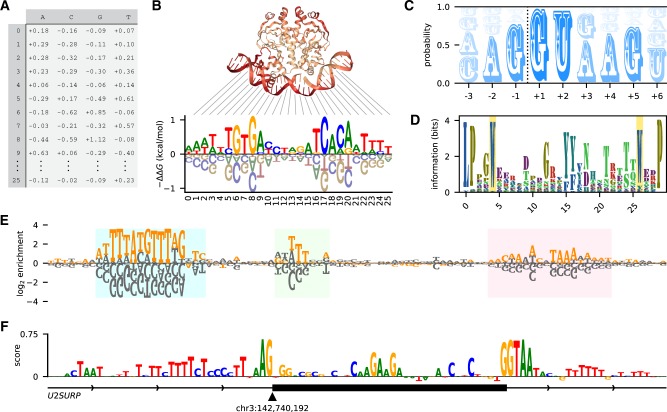
Logomaker logos can represent diverse types of data. (**A**) Example input to Logomaker. Shown is an energy matrix for the transcription factor CRP; the elements of this pandas DataFrame represent - ΔΔ*G* values contributed by each possible base (columns) at each nucleotide position (rows). Data are from Kinney *et al.* (2010). (**B**) An energy logo for CRP created by passing the DataFrame in panel A to Logomaker. The structural context of each nucleotide position is indicated [PDB 1CGP (Parkinson *et al.*, 1996)]. (**C**) A probability logo computed from all annotated 5′ splices sites in the human genome (Frankish *et al.*, 2019). The dashed line indicates the exon/intron boundary. (**D**) An information logo computed from a multiple alignment of WW domain sequences [PFAM RP15 (Finn *et al.*, 2014)], with the eponymous positions of this domain highlighted. (**E**) An enrichment logo representing the effects of mutations within the ARS1 replication origin of *S.cerevisiae*. Orange characters indicate the ARS1 wild-type sequence; highlighted regions correspond (from left to right) to the A, B1 and B2 elements of this sequence (Rao and Stillman, 1995). Data (unpublished; collected by J.B.K.) are from a mutARS-seq experiment analogous to the one reported by Liachko *et al.* (2013). (**F**) A masked logo (Shrikumar *et al.*, 2017) representing the importance scores of nucleotides in the vicinity of *U2SURP* exon 9, as predicted by a deep neural network model of splice site selection. Logo adapted (with permission) from Fig. 1D of Jaganathan *et al.* (2019). The script used to make this figure is posted on the Logomaker GitHub page at logomaker/examples/figure.ipynb

A substantial number of software tools for generating sequence logos have been described (Bailey *et al.*, 2009; Colaert *et al.*, 2009; Crooks *et al.*, 2004; Gorodkin *et al.*, 1997; Maddelein *et al.*, 2015; Menzel *et al.*, 2012; Nettling *et al.*, 2015; Olsen *et al.*, 2013; O’Shea *et al.*, 2013; Ou *et al.*, 2018; Rapin *et al.*, 2010; Schuster-Böckler *et al.*, 2004; Sharma *et al.*, 2012; Thomsen and Nielsen, 2012; Waese *et al.*, 2016; Wheeler *et al.*, 2014; Workman *et al.*, 2005; Wu and Bartel, 2017; Ye *et al.*, 2017; Yu *et al.*, 2015). However, each of these tools substantially limits the kinds of logos that one can make and the ways in which those logos can be styled. For example, WebLogo (Crooks *et al.*, 2004) was one of the first logo-generating tools to be described and is still perhaps the most widely used. WebLogo allows users to create two standard types of sequence logos (information logos and probability logos) from a list of input sequences. However, it does not allow one to generate logos from arbitrary matrices of character heights. This capability is needed for illustrating the ΔΔ*G* values of energy matrix models (Fig. 1B), the log-enrichment values obtained in high-throughput selection experiments (Fig. 1E) or importance scores that describe the predictions of deep neural networks (Fig. 1F). Moreover, although WebLogo is available as a Python package, the graphics it generates are written directly to file. This prevents logos from being customized using the matplotlib routines familiar to most Python users, or automatically incorporated into multi-panel figures.

In contrast to WebLogo and the other tools described above, ggseqlogo (Wagih, 2017) enables the creation of sequence logos within the R programming environment from arbitrary user-provided data. Importantly, ggseqlogo renders logos using native vector graphics, which facilitates *post-hoc* styling and the incorporation of logos into multi-panel figures. However, similar software is not yet available in Python. Because many biological data analysis pipelines are written in Python, there is a clear need for such logo-generating capabilities. Here we describe Logomaker, a Python package that addresses this need.

## 2 Implementation

Logomaker is a flexible Python API for creating sequence logos. Logomaker takes a pandas DataFrame as input, one in which columns represent characters, rows represent positions and values represent character heights (Fig. 1A). This enables the creation of logos for any type of data that are amenable to such a representation. The resulting logo is drawn using vector graphics embedded within a standard matplotlib Axes object, thus facilitating a high level of customization as well as incorporation into complex figures. Indeed, the logos in Figure 1 were generated as part of a single multi-panel matplotlib figure. Logomaker provides a variety of options for styling the characters within a logo, including the choice of font, color scheme, vertical and horizontal padding, etc. Logomaker also enables the highlighting of specific sequences within a logo (Fig. 1E), as well as the use of value-specific transparency in logos that illustrate probabilities (Fig. 1C). If desired, users can further customize individual characters within any rendered logo.

Because sequence logos are still commonly used to represent the statistics of multiple-sequence alignments, Logomaker provides methods for processing such alignments into matrices that can then be used to generate logos. Multiple types of matrices can be generated in this way, including matrices that represent probabilities (Fig. 1C), log odds ratios (Fig. 1E) or the information values described by Schneider and Stephens (1990) (Fig. 1D). Methods for transforming between these types of matrices are also provided. Finally, Logomaker supports the creation of masked matrices and logos that, e.g., represent deep neural network importance scores (Shrikumar *et al.*, 2017), as in Figure 1F.

## 3 Conclusion

Logomaker thus fills a major need in the Python community for flexible logo-generating software. Indeed, Logomaker has already been used to generate logos for multiple preprints and publications (Belliveau *et al.*, 2018; Barnes *et al.*, 2019; Forcier *et al.*, 2018; Kinney and McCandlish, 2019; Mason *et al.*, 2019; Nguyen *et al.*, 2019; Wong *et al.*, 2018). Logomaker is thoroughly tested, has minimal dependencies and can be installed from PyPI by executing ‘pip install logomaker’ at the command line. A step-by-step tutorial on how to use Logomaker, as well as comprehensive documentation, is available at http://logomaker.readthedocs.io.
